# Probenecid Inhibits Human Metapneumovirus (HMPV) Replication In Vitro and in BALB/c Mice

**DOI:** 10.3390/v16071087

**Published:** 2024-07-06

**Authors:** Harrison C. Bergeron, Jackelyn Crabtree, Tamas Nagy, David E. Martin, Ralph A. Tripp

**Affiliations:** 1 Department of Infectious Diseases, University of Georgia, Athens, GA 30605, USA; h.c.bergeron@uga.edu (H.C.B.);; 2 Department of Pathology, University of Georgia, Athens, GA 30605, USA; 3 TrippBio, Inc., Jacksonville, FL 32256, USA; davidmartin@trippbio.com

**Keywords:** probenecid, HMPV, antiviral, host directed, repurposed

## Abstract

Human metapneumovirus (HMPV) is an important cause of acute respiratory tract infection and causes significant morbidity and mortality. There is no specific antiviral drug to treat HMPV or vaccine to prevent HMPV. This study determined if probenecid, a host-targeting antiviral drug, had prophylactic (pre-virus) or therapeutic (post-virus) efficacy to inhibit HMPV replication in LLC-MK2 cells in vitro and in the lungs of BALB/c mice. This study showed that ≥0.5 μM probenecid significantly inhibited HMPV replication in vitro, and 2–200 mg/kg probenecid prophylaxis or treatment reduced HMPV replication in BALB/c mice.

## 1. Introduction

HMPV is an enveloped negative-sense, single-stranded, non-segmented RNA virus and a member of the *Pneumoviridae* family [1]. HMPV consists of groups A and B, each divided into subclasses consisting of A1, A2, B1, and B2, each with year-to-year variability [2]. HMPV has seasonal distribution affecting younger children, with infants less than 12 months of age at a higher risk of severe infection [3]. Infection with HMPV is estimated to cause >14 million acute lower respiratory infections in those under 5 years of age and as many as 61,000 infant deaths per year [4]. Thus, infections of HMPV are life-threatening in the young but usually cause mild symptoms in healthy adults [5]. Similar to respiratory syncytial virus (RSV), influenza virus (flu), and seasonal coronaviruses (CoV), HMPV can cause clinical symptoms that include fever, cough, and wheezing, and co-infections with HMPV are common, i.e., ranging from 3% to 11% [6]. Despite the disease burden imposed by HMPV, no safe and effective vaccines or treatments exist; thus, the mainstay of treatment is supportive. Many of the antiviral treatments investigated for RSV have also been examined for HMPV, including the nucleoside analog ribavirin [7], viral fusion inhibitors [8], monoclonal antibodies [9,10], and others. 

Probenecid, belonging to the uricosurics class of drugs, is a safe FDA-approved drug that has been used to lower urate levels associated with gout for >6 decades [11]. Our lab has shown that probenecid has potent nano- to micromolar antiviral drug activity when administered either prophylactically (pre-virus infection) or therapeutically (post-virus infection) and potently inhibits the replication of RSV variants [12], influenza virus types and strains [13,14], and SARS-CoV-2 variants [15] in respiratory epithelial (A549) cells, primary normal human bronchial epithelial (NHBE) cells, and mouse and hamster models of infection. In addition, probenecid has anti-inflammatory qualities, reducing several pro-inflammatory cytokines such as IL-6, TNF-α, and IL-1β [15,16]. Importantly, probenecid treatment was very effective in a Phase 2 randomized, placebo-controlled, single-blind, dose range-finding study in non-hospitalized patients with symptomatic, mild-to-moderate COVID-19 [17]. In this clinical study, the median time to viral clearance was significantly shorter in patients receiving 1000 mg probenecid compared to placebo (7 vs. 11 days, respectively; *p* < 0.0001), and for patients receiving 500 mg probenecid versus placebo, it was 9 vs. 11 days, respectively (*p* < 0.0001). In addition, the median time to viral clearance was significantly shorter for the probenecid 1000 mg group than for the probenecid 500 mg group (7 vs. 9 days, respectively; *p* < 0.0001). 

The antiviral activities of probenecid were first revealed in RNA interference (RNAi) screens of human A549 cells infected with influenza A viruses (IAVs) that were functionally screened for druggable host genes required for IAV replication [18,19,20,21,22]. One of the host genes discovered from the siRNA screens was the OAT3 gene, which is known to be involved in the transport and excretion of organic ions and metabolites [13]. To determine the broadly efficacious antiviral mechanism that appeared common to the various viruses inhibited by probenecid [12,13,14,15,17,23], we examined if mitogen-activated protein kinases (MAPKs) were involved because the MAPK pathway was used in the replication of these viruses [24]. We examined the role of probenecid treatment on the extracellular signal-regulated kinase (ERK) and c-Jun N-terminal kinase (JNK) downstream pathways affecting virus replication in A549 cells. We found that probenecid inhibits the RSV-induced phosphorylation of JNK and ERK and the downstream phosphorylation of c-jun, a component of the AP-1 transcription complex needed for virus replication (manuscript submitted). The inhibition of JNK by probenecid also reversed the repression of transcription factor hepatocyte nuclear factor-4 (HNF-4). Thus, the probenecid inhibition of JNK and ERK phosphorylation involved a pathway that precludes virus replication. Evidence indicates that JNK regulates many cellular processes and is activated during viral infections [25,26]. When JNK is activated by the sequential phosphorylation of upstream kinases, it translocates from the cytoplasm to the nucleus [27,28], where it can regulate transcription factors such as HNF-4 [29], which regulates the expression of OATs [30,31]. Thus, it appears that the mechanism of action of probenecid inhibition is linked to the inhibition of JNK phosphorylation and downstream HNF-4 expression, which regulates OAT3 gene expression, preventing virus assembly/replication.

Since probenecid treatment had prophylactic (pre-virus) and therapeutic (post-virus) efficacy to inhibit RSV replication in Vero E6 cells, HEp-2 cells, NHBE cells, and BALB/c mice [12], and HMPV and RSV are closely related viruses causing similar respiratory tract infections [32], we evaluated the prophylactic and therapeutic efficacy of probenecid for inhibiting HMPV/CAN/97-83 (CAN83) replication in vitro and in mice. 

## 2. Materials and Methods

### 2.1. Cell Culture

LLC-MK2 cells (ATCC; CCL7), an immortalized Rhesus macaque kidney cell line, were grown in OptiMEM (Gibco, ThermoFisher, Waltham, MA, USA) supplemented with 10% heat-inactivated fetal bovine serum (HyClone, Logan, UT, USA). The cells were maintained in the log phase at 37 °C in a 5% CO_2_ incubator and used to propagate CAN83 and for plaque assays.

### 2.2. Human Metapneumovirus (HMPV) 

The A2 sub-lineage, CAN83, originally isolated in Canada from a clinical sample [33], was propagated and quantified in LLC-MK2 cells as described [34]. Briefly, cells were grown to 80% confluence in Opti-MEM (Gibco) supplemented with 10% heat-inactivated fetal bovine serum (HyClone). For virus infection, cells were washed with Dulbecco’s phosphate-buffered saline (DPBS; Gibco) and then infected with HMPV (MOI = 0.1) for 1 h at room temperature to allow for virus adsorption. Following this, Opti-MEM supplemented with 1 μg/mL TPCK trypsin (Worthington) and 100 µg/mL CaCl_2_ (Gibco) was added to the cells and incubated for 5 days before harvesting the virus. For virus harvesting, the infected cells were scraped, and the lysates were transferred to a 50 mL tube and sonicated 3 times for 1 min. The material was clarified by centrifugation (3000 RPM for 10 min), and the supernatant containing the HMPV was aliquoted and flash-frozen for later use, as previously described [9]. 

### 2.3. HMPV Plaque Assay

Viral titers were determined by plaque assay, as previously described [35]. LLC-MK2 cells were plated on a 24-well plate (Costar, Corning, NY, USA) to achieve 100% confluency. Cells were washed in Hanks Balanced Salt Solution (HBSS, Sigma), and 10-fold dilutions of lung homogenate or cell supernatant were made in OptiMEM (Gibco) supplemented with 1 μg/mL TPCK trypsin (ThermoFisher). Virus dilutions were incubated for 2 h, followed by overlay with 2% methylcellulose (ThermoFisher) supplemented with 1 μg/mL TPCK trypsin without FBS. After 6 days, overlay media were removed, and the cells were washed in HBSS and fixed with 60:40 acetone–methanol (Sigma) for 20 min at room temperature. After fixation, plates were washed 3× with KPL wash buffer (Seracare, Milford, MA, USA), blocked with Blotto (5% non-fat dry milk), then incubated for 60 min with a human anti-HMPV antibody (clone MPV346; provided by Jarrod Mousa, UGA), subsequently washed with HBSS, and then followed by a 45 min incubation with secondary goat anti-human-AP (Fisher). Plaques were developed with NBT/BCIP (Sigma), rinsed with deionized water, and enumerated using a dissection microscope (Zeiss, Cambridge, UK). 

### 2.4. Probenecid Treatment and HMPV Replication

A working stock of probenecid (CAS Number: 57-66-9; Sigma, St. Louis, MO, USA) was dissolved in DMSO (Sigma), and dilutions from a 100 mM working stock were made. For prophylactic or therapeutic treatment, LLC-MK2 cells were plated overnight at 2.5 × 10^4^ cells/well in 96-well flat-bottom plates (Costar, ThermoFisher, Waltham, MA, USA). Cells were pre-treated prophylactically for 24 h before infection or therapeutically treated at 1 h post-infection with probenecid at different concentrations, i.e., 100, 50, 25, 12, 6, 3, 1, 0.5, 0.2, 0.1, and 0 µM. LLC-MK2 cells were plated in 24-well plates (Corning, Corning, NY, USA). For the prophylactically treated cells, the tissue culture was decanted, the cells were washed with PBS (Gibco), and probenecid in complete tissue culture media was added to the wells and incubated for 24 h. For both the prophylactic and therapeutic treatment groups, CAN83 diluted with serum-free media with 1 μg/mL TPCK trypsin was added to the wells at an MOI of 0.1. The infection was incubated for 1 h at 37 °C, after which CAN83 was removed, probenecid was added to the respective wells in overlay media, and the assay continued at 37 °C for 6 days. HMPV plaques were stained and enumerated as described above. 

### 2.5. Mice 

Female BALB/c mice that were 6–8 weeks old were purchased from Charles River Laboratory (Raleigh, NC, USA) and rested for a week before use. The Institutional Review Board of the University of Georgia, A2021 03-006-Y2-A0, approved the animal use protocol, Immunity to Respiratory Viruses and Virus Proteins in Mus Musculus, 6 May 2021. To determine the efficacy of probenecid on HMPV replication, the mice were orally gavaged (p.o.) either 24 h pre-virus (prophylactic) or 24 h post-virus (therapeutic) with either 2 or 200 mg/kg probenecid diluted in PBS or given PBS alone (n = 5 mice/group). Avertin (2,2,2-tribromoethanol; Sigma)-anesthetized mice were intranasally (i.n.) infected with 10^6^ PFU CAN83 diluted in PBS. On day 5 pi, the mice were euthanized by Avertin, then cervically dislocated and exsanguinated. To determine lung viral loads, lungs were removed and stored on ice in gentleMACS tubes containing 1 mL OptiMEM, then homogenized using a gentleMACS tissue dissociator (Miltenyi Biotec, Waltham, MA, USA), centrifuged at 200 xG, and the supernatants containing the virus were used immediately in the plaque assay described above. 

### 2.6. BAL Cytokines and Chemokines

As described above, bronchoalveolar lavages (BALs) were collected from Avertin-euthanized mice. The trachea was exposed, a small incision was made, and an 18-gauge catheter was inserted. The lungs were flushed 3× with 1 mL 0.8% BSA/1× Halt™ protease inhibitor cocktail (ThermoFisher) diluted in PBS and collected in 1.5 mL snap-cap tubes. The BALs were centrifuged for 10 min at 500 xG at 4 °C, and the supernatant (BAL fluid, BALF) was separated and stored at −80 °C until cytokine/chemokine analysis. BALF was used in the Milliplex MAP Mouse Cytokine/Chemokine Immunology 25-plex Assay as described by the manufacturer (Millipore Sigma, Rockville, MD, USA) using standards and quality controls included in the kit. Individual BALF samples were duplicated from n = 3 mice/group in one experiment. Samples were analyzed on a Luminex 200 instrument (Luminex Corporation, Austin, TX, USA) using Luminex xPONENT 3.1 software. 

### 2.7. Statistical Analysis 

Statistical analyses were performed using a one-way analysis of variance (ANOVA), as indicated in the figure legends. Representative data from at least two independent experiments are shown. Data were analyzed for statistical significance where *p* < 0.05 was considered statistically significant using Prism 9 (GraphPad). The results were calculated and presented as means +/− standard error.

## 3. Results

### 3.1. Probenecid Inhibits HMPV In Vitro 

**Figure 1:**
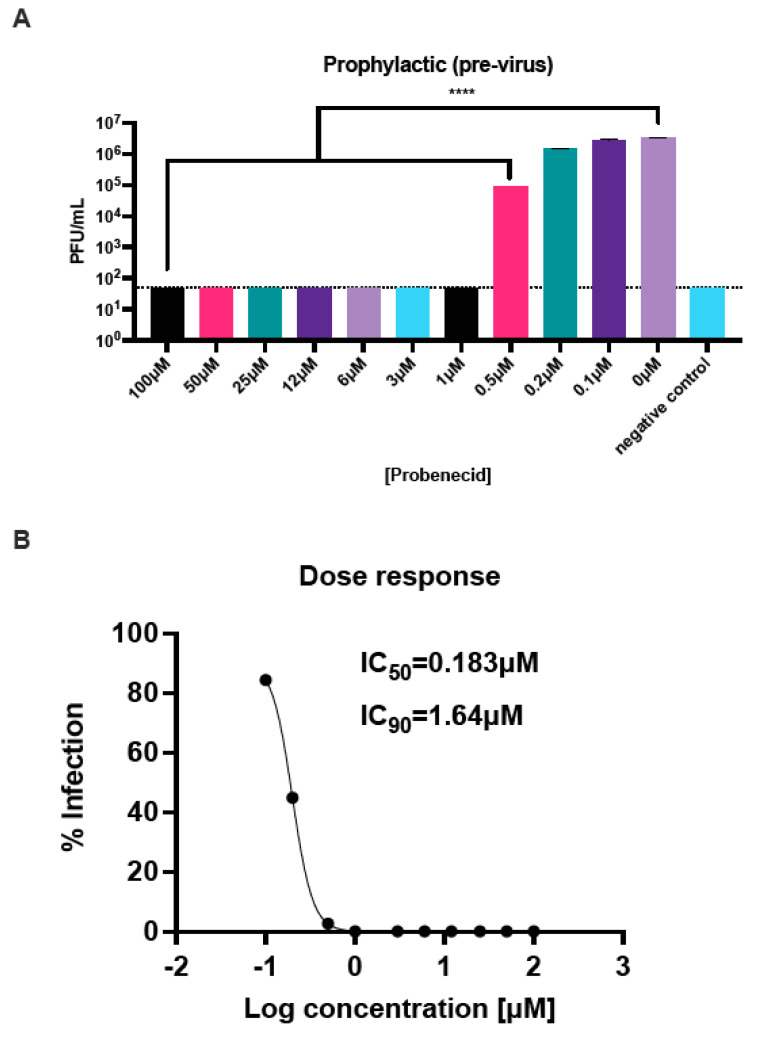
LLC-MK2 cells prophylactically treated with probenecid reduce HMPV replication. LLC-MK2 cells were treated with the indicated concentrations of probenecid for 24 h. After 24 h of pre-treatment, cells were infected at an MOI = 0.1 of CAN83 and incubated for 1 h. After incubation, the infection was removed, the cells were washed, and the media decanted. Media containing probenecid were added to the cells for 24 h. Plaques were enumerated, and (**A**) titers and (**B**) dose–response curves were determined. Probenecid prophylaxis was significantly (**** *p* < 0.0001) effective at concentrations ≥ 0.5 μM. These data represent three independent experiments, each with three experimental replicates. All statistics are one-way ANOVA with Dunnett’s multiple comparison correction, with comparisons performed against infected cells.

**Figure 2:**
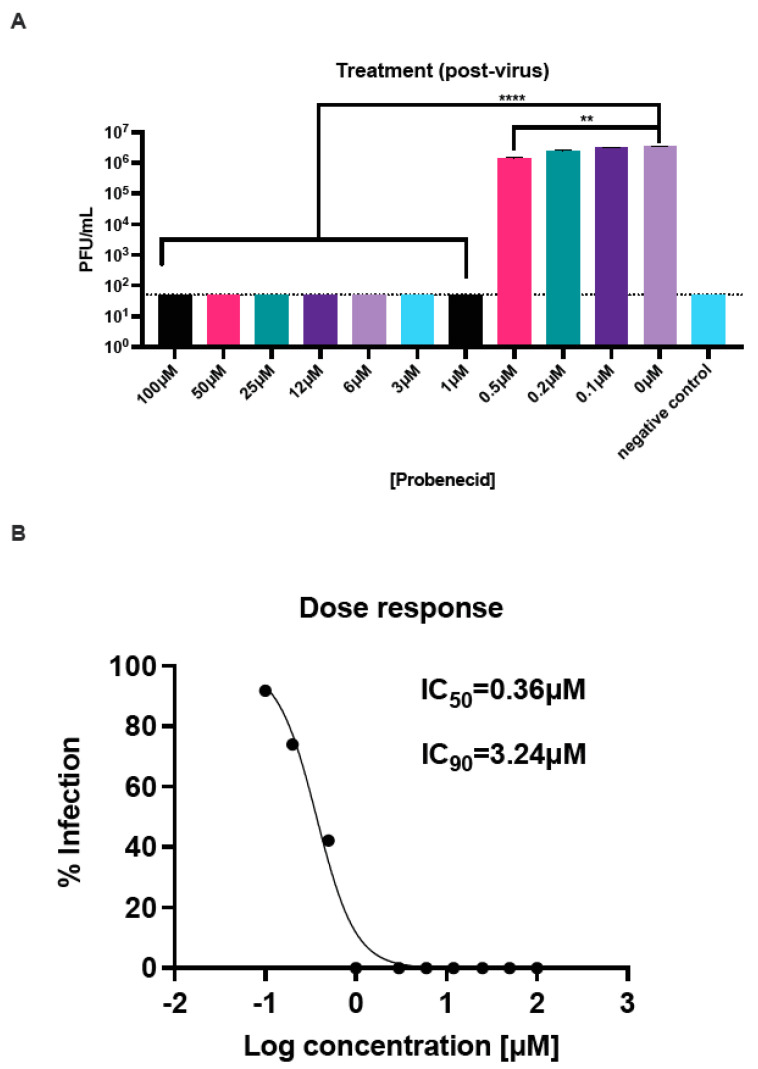
LLC-MK2 cells therapeutically treated with probenecid reduce HMPV replication. LLC-MK2 cells were infected with CAN83 at an MOI of 0.1 and incubated for 1 h. After incubation, the infection was removed, the cells were washed, and the media decanted. Media containing probenecid at the indicated concentrations were added to the cells. Plaques were enumerated, and (**A**) titers and (**B**) dose–response curves were determined. Probenecid treatment was significantly (** *p* < 0.01, **** *p* < 0.0001) effective at concentrations ≥ 0.5 μM. These data represent three independent experiments, each with three experimental replicates. All statistics are one-way ANOVA with Dunnett’s multiple comparison correction, with comparisons performed against infected cells.

LLC-MK2 cells support HMPV replication [36]. Thus, we examined if probenecid treatment inhibited virus replication either 24 h pre-virus (prophylactic) (Figure 1) or 1 h post-virus (therapeutic) (Figure 2) infection (MOI = 0.1) with CAN83. The results show that pre- and post-virus treatment with probenecid potently inhibited HMPV replication. The HMPV infection of LLC-MK2 cells was significantly (*p* < 0.0001) reduced in cells treated with probenecid at concentrations ≥ 0.5μM prophylactically (Figure 1A) and therapeutically (Figure 2A). At doses ≥ 1.0 μM, the plaque assay did not detect HMPV. For pre-virus prophylactic treatment, the IC_50_ was calculated to be 0.18 μM, and the IC_90_ was 1.64 μM following infection with CAN83 (Figure 1B). The IC_50_ was 0.36 μM for therapeutic post-virus treatment, and the IC_90_ was 3.24 μM (Figure 2B). We recently showed that probenecid was highly effective at inhibiting RSV strain A and B replication in vitro [12]. Specifically, probenecid prophylaxis resulted in a dose-dependent decrease in RSV A2 replication where the IC_50_/IC_90_ was 0.07/0.63 μM in Vero E6 cells, 0.8/7.2 μM in HEp-2 cells, and 0.4/3.6 μM in NHBE cells [12]. These results show that the potent inhibitory effects of probenecid on HMPV replication in LLC-MK2 cells are consistent with our previous RSV studies. 

### 3.2. Probenecid Inhibits HMPV In Vivo 

**Figure 3:**
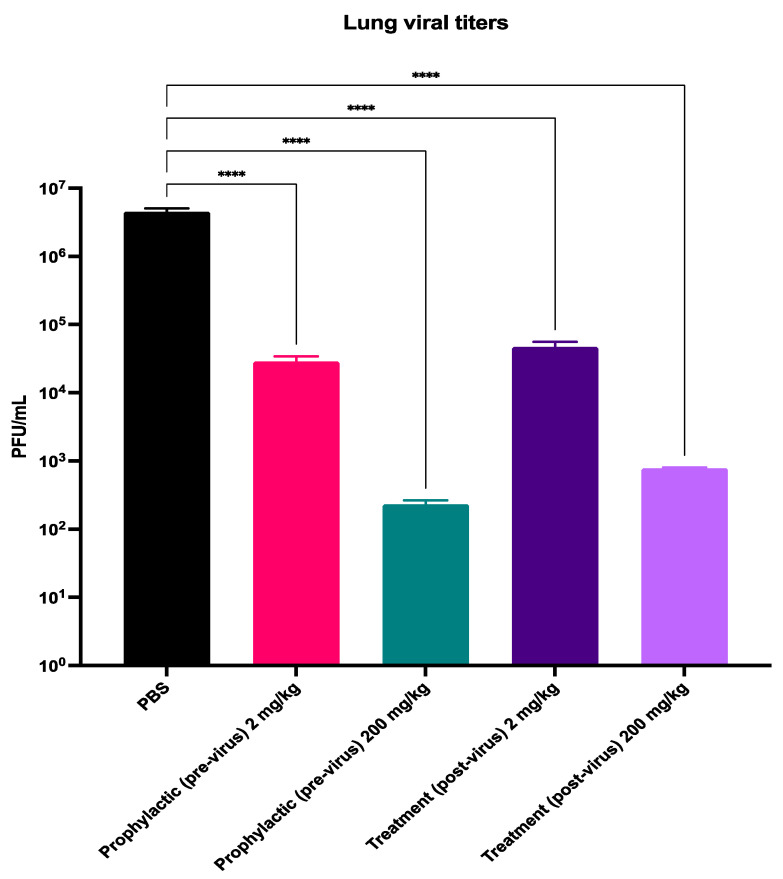
Probenecid reduces HMPV replication in mice. Female BALB/c mice were treated with 2 or 200 mg/kg probenecid or PBS either 24 h prior to (prophylactic) or 24 h post- (treatment) infection with 10^6^ PFU CAN83. At least two independent repeats were performed with representative data shown. Bars represent mean PFU/mL +/− SEM of n = 3–5 mice/group. **** *p* < 0.0001 compared to PBS-treated mice, as determined by one-way ANOVA.

Additionally, we previously showed that mice treated once with probenecid at 2 or 200 mg/kg, either 24 h pre-virus (prophylaxis) or 24 h post-virus (treatment), had considerable reductions in RSV lung virus titers [12]. Given the similarity of HMPV to RSV in epidemiology and infection [32,37], we determined if probenecid could inhibit HMPV replication in BALB/c mice. BALB/c mice are commonly used in RSV and HMPV studies to evaluate drugs and vaccines. These mice are semi-permissive to these viruses and are replacement animal models for chimpanzees and other non-human primates (NHPs) [38,39]. Thus, BALB/c mice were treated (p.o.) with either 2 or 200 mg/kg probenecid or PBS, either 24 h pre-virus (prophylaxis) or 24 h post-virus (treatment). Mice were i.n. infected with 10^6^ PFU CAN83 as described in the Methods. At the peak of lung viral replication, i.e., day 5 pi, the mice were sacrificed, and lungs were collected to determine the HMPV titers and histopathology, as previously described [9,40]. Figure 3 shows that probenecid treatment before or after CAN83 infection significantly (*p* < 0.0001) reduced lung viral titers in mice compared to untreated mice. These dose-dependent reductions supported our in vitro results (Figure 1 and Figure 2) and previous in vivo studies of other viruses [12,13,15]. Regardless of treatment timing, mice treated with 2 mg/kg probenecid had a ~2 log reduction in lung viral titers, and mice dosed with 200 mg/kg probenecid had a ~4 log reduction. These data support the use of probenecid as a potential prophylactic or therapeutic treatment for HMPV in vivo. 

### 3.3. Immune Profiling during HMPV Challenge and Probenecid Treatment

**Figure 4:**
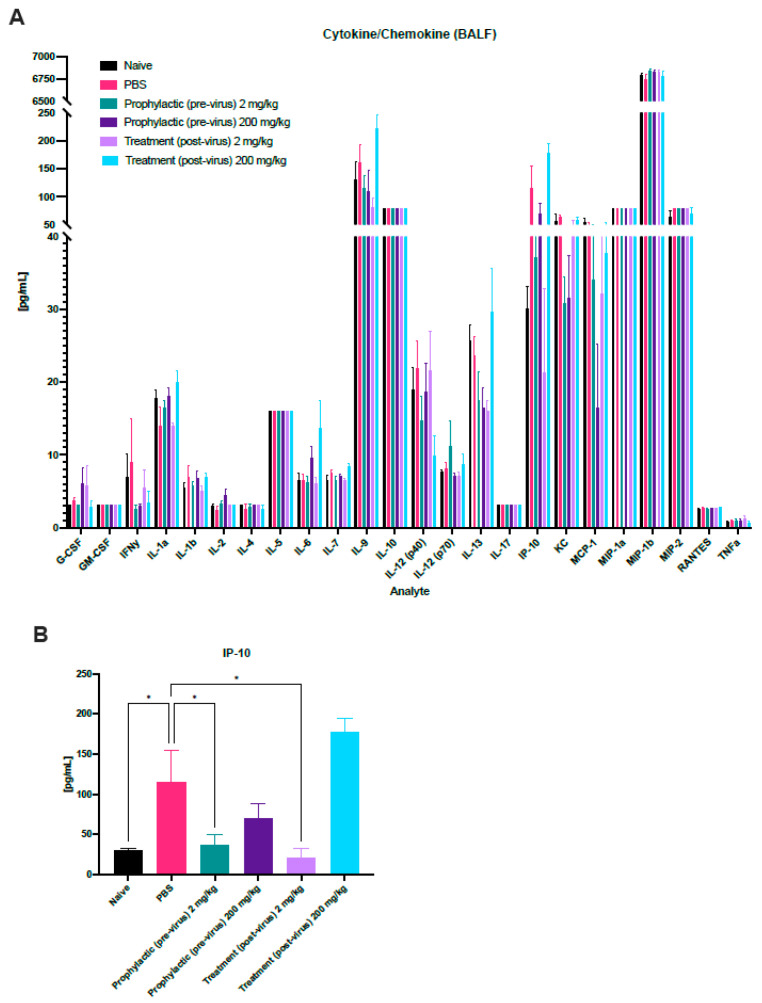
Cytokine and chemokine responses during HMPV infection. Female BALB/c mice (n = 3/group) were treated with 2 or 200 mg/kg probenecid or PBS either 24 h prior to (prophylactic) or 24 h post- (treatment) infection with 10^6^ PFU CAN83. BALF was collected on day 5 pi, and samples were analyzed in duplicate by a Luminex multiplex cytokine/chemokine (25-plex) kit. (**A**) Immune responses were quantified, and most analytes remained unchanged regardless of infection or treatment. (**B**) IP-10 concentrations are shown. Each analyte was analyzed by a one-way ANOVA, compared to PBS-treated mice, where * *p* ≤ 0.05.

To evaluate how probenecid treatment may affect the cytokine responses to HMPV infection, BALF was collected on day 5 pi and subjected to cytokine and chemokine analysis by a 25-plex Luminex kit. Overall, HMPV infection did not drastically modify cytokine responses in the BALF of BALB/c mice at this time (Figure 4A). Of the 25 analytes interrogated, only IP-10 (Figure 4B) was significantly modified during HMPV infection. Compared to untreated, uninfected mice, PBS-treated, infected mice had significantly (*p* < 0.05) increased levels of IP-10 in the BALF. Pre-virus and post-virus treatment with 2 mg/kg probenecid significantly affected IP-10 levels in the BALF, but interestingly, 200 mg/kg did not significantly reduce IP-10. 

## 4. Discussion

HMPV causes respiratory tract infections in children, adults, the elderly, and immunocompromised patients [41]. The predominant clinical features caused by HMPV infection are upper and lower respiratory tract infections that can lead to pneumonia and bronchiolitis [41]. There are no licensed antiviral drugs or vaccines approved against HMPV. However, vaccine candidates are in the preclinical research stage, and numerous neutralizing monoclonal antibodies against HMPV have been characterized together with their targeting epitopes [9,42,43,44,45]. Infection with HMPV is similar to RSV, where infection typically occurs in the young, with reinfection occurring throughout life [46]. The treatment of HMPV infection is typically supportive, where medications including over-the-counter analgesics and decongestants are used to control pain, fever, and congestion. Currently, novel therapeutic strategies against HMPV have focused on broad-spectrum antiviral drug repurposing and HMPV F protein-direct strategies [47]. Examples include intravenous immunoglobulins (IVIG) made of polyclonal antibodies against HMPV, ribavirin, and other nucleotide analogs (e.g., favipiravir), viral attachment inhibitors (e.g., DAS181), and F protein inhibitors (e.g., synthetic peptides against the HRA and HRB domains of the HMPV F protein). Without an antiviral treatment or vaccine, HMPV infection remains an important burden on the healthcare system. Thus, we sought to determine if probenecid treatment could inhibit HMPV replication. 

Probenecid is FDA-approved and has been used safely for decades to treat gout and inflammatory arthritis by facilitating the excretion of uric acid [48]. Using RNA interference (RNAi) screens to determine the host genes required for influenza A virus (IAV) replication in human respiratory epithelial cells, i.e., A549 cells, we showed that the organic anion transporter, OAT3, was required for IAV replication [13,20,21]. OAT3 is a druggable host gene, and probenecid is a pharmacological inhibitor of OAT3 [13,49]. Further, probenecid has a benign clinical safety profile [50]. Thus, we explored the antiviral features of probenecid and showed that it is a broadly efficacious antiviral drug with pico- to micromolar inhibitory in vitro and in vivo activity for IAV strains and highly pathogenic influenza viruses [13,23], SARS-CoV-2 variants [17,50], and RSV strains [12,50]. We also showed that probenecid reduced aspects of the pro-inflammatory response [16,51]. A recent study did not repeat our results for SARS-CoV-2 [52]. It should be noted there were several differences between the studies. Specifically, different drug concentrations were tested in vitro and in vivo, different virus variants were compared, different cell lines were compared, and different readouts of drug efficacy were measured. We measured virus replication by PFU versus Box et al. who evaluated cytopathic activity (cell death) and performed PCR, which cannot be used to measure virus replication as PCR primers will detect DNA fragments and produce false positives. Further, there were differences in the duration of in vitro experiments and with the probenecid concentrations used. 

Consistent with our previous work on other respiratory viruses [12,13,15], we report the potential of probenecid to regulate HMPV replication. We show that low micromolar (high nanomolar) treatment of LLC-MK2 cells with probenecid reduces HMPV replication. At concentrations ≥ 1.0 μM, no HMPV was recovered in either pre-virus or post-virus-treated cells. Similarly, mice treated with 200 mg/kg at 24 h pre- or post-HMPV infection had substantial (log) reductions in lung viral titers. The BALF cytokine results did not show robust changes associated with CAN83 infection, which is consistent with the lack of lung pathology observed. No remarkable histopathology in HMPV-infected mice was detected by the H&E staining of lung sections, so we could not determine if probenecid treatment affected lung damage. Various studies have reported differential pathology and immune responses to HMPV in the mouse model, which is likely dependent on strain, passage number, and inoculum [53,54,55]. Despite this limitation, our results support using probenecid as an effective antiviral drug and further expand the virus repertoire susceptible to probenecid treatment. Future studies should investigate low-passaged clinical isolates of HMPV known to induce stronger immune responses and histopathology in vivo.

Given the substantial reduction in HMPV replication in vitro and in vivo and the antiviral effects on several other respiratory pathogens, including influenza, RSV, and SARS-CoV-2 [14,50], the mechanism of action is likely shared among these respiratory viruses. We recently showed that probenecid inhibits the phosphorylation of JNK and ERK and the downstream phosphorylation of c-jun, which is needed for virus replication (manuscript submitted). Thus, the probenecid inhibition of JNK and ERK phosphorylation involves a pathway that precludes virus replication and could be a broadly antiviral mechanism. There are benefits of broadly effective antiviral drugs other than the obvious as they may be used to circumvent the need for some diagnostics. For example, patients with viral respiratory disease may be treated with a broadly active drug like probenecid, regardless of the specific pathogen causing the disease. This may be limited by the range of drug efficacy, but probenecid is safe and has been used in humans for >6 decades as a treatment for gout. 

## Data Availability

The data presented in this study are available on request from the corresponding author.

## References

[1] Kumar P., Srivastava M. (2018). Prophylactic and therapeutic approaches for human metapneumovirus. Virusdisease.

[2] Uddin S., Thomas M. (2024). Human Metapneumovirus.

[3] Rodriguez P.E., Frutos M.C., Adamo M.P., Cuffini C., Camara J.A., Paglini M.G., Moreno L., Camara A. (2020). Human Metapneumovirus: Epidemiology and genotype diversity in children and adult patients with respiratory infection in Cordoba, Argentina. PLoS ONE.

[4] Wang X., Li Y., Deloria-Knoll M., Madhi S.A., Cohen C., Ali A., Basnet S., Bassat Q., Brooks W.A., Chittaganpitch M. (2021). Global burden of acute lower respiratory infection associated with human metapneumovirus in children under 5 years in 2018: A systematic review and modelling study. Lancet Glob Health.

[5] Esposito S., Mastrolia M.V. (2016). Metapneumovirus Infections and Respiratory Complications. Semin. Respir. Crit. Care Med..

[6] Kinder J.T., Moncman C.L., Barrett C., Jin H., Kallewaard N., Dutch R.E. (2020). Respiratory Syncytial Virus and Human Metapneumovirus Infections in Three-Dimensional Human Airway Tissues Expose an Interesting Dichotomy in Viral Replication, Spread, and Inhibition by Neutralizing Antibodies. J. Virol..

[7] Chu H.Y., Renaud C., Ficken E., Thomson B., Kuypers J., Englund J.A. (2014). Respiratory Tract Infections Due to Human Metapneumovirus in Immunocompromised Children. J. Pediatric Infect. Dis. Soc..

[8] Chang A., Masante C., Buchholz U.J., Dutch R.E. (2012). Human metapneumovirus (HMPV) binding and infection are mediated by interactions between the HMPV fusion protein and heparan sulfate. J. Virol..

[9] Banerjee A., Huang J., Rush S.A., Murray J., Gingerich A.D., Royer F., Hsieh C.L., Tripp R.A., McLellan J.S., Mousa J.J. (2022). Structural basis for ultrapotent antibody-mediated neutralization of human metapneumovirus. Proc. Natl. Acad. Sci. USA.

[10] Cseke G., Wright D.W., Tollefson S.J., Johnson J.E., Crowe J.E., Williams J.V. (2007). Human metapneumovirus fusion protein vaccines that are immunogenic and protective in cotton rats. J. Virol..

[11] Garcia-Rodriguez C., Mujica P., Illanes-Gonzalez J., Lopez A., Vargas C., Saez J.C., Gonzalez-Jamett A., Ardiles A.O. (2023). Probenecid, an Old Drug with Potential New Uses for Central Nervous System Disorders and Neuroinflammation. Biomedicines.

[12] Murray J., Bergeron H.C., Jones L.P., Reener Z.B., Martin D.E., Sancilio F.D., Tripp R.A. (2022). Probenecid Inhibits Respiratory Syncytial Virus (RSV) Replication. Viruses.

[13] Perwitasari O., Yan X., Johnson S., White C., Brooks P., Tompkins S.M., Tripp R.A. (2013). Targeting organic anion transporter 3 with probenecid as a novel anti-influenza a virus strategy. Antimicrob. Agents Chemother..

[14] Murray J., Martin D.E., Hosking S., Orr-Burks N., Hogan R.J., Tripp R.A. (2024). Probenecid Inhibits Influenza A(H5N1) and A(H7N9) Viruses In Vitro and in Mice. Viruses.

[15] Murray J., Hogan R.J., Martin D.E., Blahunka K., Sancilio F.D., Balyan R., Lovern M., Still R., Tripp R.A. (2021). Probenecid inhibits SARS-CoV-2 replication in vivo and in vitro. Sci. Rep..

[16] Rosli S., Kirby F.J., Lawlor K.E., Rainczuk K., Drummond G.R., Mansell A., Tate M.D. (2019). Repurposing drugs targeting the P2X7 receptor to limit hyperinflammation and disease during influenza virus infection. Br. J. Pharmacol..

[17] Martin D.E., Pandey N., Chavda P., Singh G., Sutariya R., Sancilio F., Tripp R.A. (2023). Oral Probenecid for Nonhospitalized Adults with Symptomatic Mild-to-Moderate COVID-19. Viruses.

[18] Tripp R.A., Mark Tompkins S. (2015). Antiviral effects of inhibiting host gene expression. Curr. Top. Microbiol. Immunol..

[19] Tripp R.A., Mejias A., Ramilo O. (2013). Host gene expression and respiratory syncytial virus infection. Curr. Top. Microbiol. Immunol..

[20] Perwitasari O., Bakre A., Tompkins S.M., Tripp R.A. (2013). siRNA Genome Screening Approaches to Therapeutic Drug Repositioning. Pharmaceuticals.

[21] Meliopoulos V.A., Andersen L.E., Birrer K.F., Simpson K.J., Lowenthal J.W., Bean A.G., Stambas J., Stewart C.R., Tompkins S.M., van Beusechem V.W. (2012). Host gene targets for novel influenza therapies elucidated by high-throughput RNA interference screens. FASEB J..

[22] Tripp R.A., Tompkins S.M. (2009). Therapeutic applications of RNAi for silencing virus replication. Methods Mol. Biol..

[23] Murray J., Martin D.E., Sancilio F.D., Tripp R.A. (2023). Antiviral Activity of Probenecid and Oseltamivir on Influenza Virus Replication. Viruses.

[24] Kumar R., Khandelwal N., Thachamvally R., Tripathi B.N., Barua S., Kashyap S.K., Maherchandani S., Kumar N. (2018). Role of MAPK/MNK1 signaling in virus replication. Virus Res..

[25] Caly L., Li H.M., Bogoyevitch M.A., Jans D.A. (2017). c-Jun N-terminal kinase activity is required for efficient respiratory syncytial virus production. Biochem. Biophys. Res. Commun..

[26] Chen J., Ye C., Wan C., Li G., Peng L., Peng Y., Fang R. (2021). The Roles of c-Jun N-Terminal Kinase (JNK) in Infectious Diseases. Int. J. Mol. Sci..

[27] Ha J., Kang E., Seo J., Cho S. (2019). Phosphorylation Dynamics of JNK Signaling: Effects of Dual-Specificity Phosphatases (DUSPs) on the JNK Pathway. Int. J. Mol. Sci..

[28] Sehgal V., Ram P.T. (2013). Network Motifs in JNK Signaling. Genes. Cancer.

[29] Lu H. (2016). Crosstalk of HNF4alpha with extracellular and intracellular signaling pathways in the regulation of hepatic metabolism of drugs and lipids. Acta Pharm. Sin. B.

[30] Li T.T., An J.X., Xu J.Y., Tuo B.G. (2019). Overview of organic anion transporters and organic anion transporter polypeptides and their roles in the liver. World J. Clin. Cases.

[31] Nigam S.K., Granados J.C. (2023). OAT, OATP, and MRP Drug Transporters and the Remote Sensing and Signaling Theory. Annu. Rev. Pharmacol. Toxicol..

[32] Akhras N., Weinberg J.B., Newton D. (2010). Human metapneumovirus and respiratory syncytial virus: Subtle differences but comparable severity. Infect. Dis. Rep..

[33] Peret T.C., Boivin G., Li Y., Couillard M., Humphrey C., Osterhaus A.D., Erdman D.D., Anderson L.J. (2002). Characterization of human metapneumoviruses isolated from patients in North America. J. Infect. Dis..

[34] Bar-Peled Y., Diaz D., Pena-Briseno A., Murray J., Huang J., Tripp R.A., Mousa J.J. (2019). A Potent Neutralizing Site III-Specific Human Antibody Neutralizes Human Metapneumovirus In Vivo. J. Virol..

[35] Xu J., Zhang Y., Williams J.V. (2018). Development and optimization of a direct plaque assay for trypsin-dependent human metapneumovirus strains. J. Virol. Methods.

[36] Deffrasnes C., Cote S., Boivin G. (2005). Analysis of replication kinetics of the human metapneumovirus in different cell lines by real-time PCR. J. Clin. Microbiol..

[37] Williams J.V., Edwards K.M., Weinberg G.A., Griffin M.R., Hall C.B., Zhu Y., Szilagyi P.G., Wang C.K., Yang C.F., Silva D. (2010). Population-based incidence of human metapneumovirus infection among hospitalized children. J. Infect. Dis..

[38] Taylor G. (2017). Animal models of respiratory syncytial virus infection. Vaccine.

[39] Schildgen O., Simon A., Williams J. (2007). Animal models for human metapneumovirus (HMPV) infections. Vet. Res..

[40] Huang J., Chopra P., Liu L., Nagy T., Murray J., Tripp R.A., Boons G.J., Mousa J.J. (2021). Structure, Immunogenicity, and Conformation-Dependent Receptor Binding of the Postfusion Human Metapneumovirus F Protein. J. Virol..

[41] Wang S.M., Liu C.C., Wang H.C., Su I.J., Wang J.R. (2006). Human metapneumovirus infection among children in Taiwan: A comparison of clinical manifestations with other virus-associated respiratory tract infections. Clin. Microbiol. Infect..

[42] Fausther-Bovendo H., Hamelin M.E., Carbonneau J., Venable M.C., Checkmahomed L., Lavoie P.O., Ouellet M.E., Boivin G., D’Aoust M.A., Kobinger G.P. (2022). A Candidate Therapeutic Monoclonal Antibody Inhibits Both HRSV and HMPV Replication in Mice. Biomedicines.

[43] Guo L., Li L., Liu L., Zhang T., Sun M. (2023). Neutralising antibodies against human metapneumovirus. Lancet Microbe.

[44] Yim K.C., Mousa J.J., Blanco J.C.G., Kim S., Boukhvalova M.S. (2023). Human Metapneumovirus (hMPV) Infection and MPV467 Treatment in Immunocompromised Cotton Rats Sigmodon hispidus. Viruses.

[45] Liu P., Shu Z., Qin X., Dou Y., Zhao Y., Zhao X. (2013). A live attenuated human metapneumovirus vaccine strain provides complete protection against homologous viral infection and cross-protection against heterologous viral infection in BALB/c mice. Clin. Vaccine Immunol..

[46] Falsey A.R., Erdman D., Anderson L.J., Walsh E.E. (2003). Human metapneumovirus infections in young and elderly adults. J. Infect. Dis..

[47] Van Den Bergh A., Bailly B., Guillon P., von Itzstein M., Dirr L. (2022). Antiviral strategies against human metapneumovirus: Targeting the fusion protein. Antivir. Res..

[48] Robbins N., Koch S.E., Tranter M., Rubinstein J. (2012). The history and future of probenecid. Cardiovasc. Toxicol..

[49] Hagos F.T., Daood M.J., Ocque J.A., Nolin T.D., Bayir H., Poloyac S.M., Kochanek P.M., Clark R.S., Empey P.E. (2017). Probenecid, an organic anion transporter 1 and 3 inhibitor, increases plasma and brain exposure of N-acetylcysteine. Xenobiotica.

[50] Tripp R.A., Martin D.E. (2022). Repurposing Probenecid to Inhibit SARS-CoV-2, Influenza Virus, and Respiratory Syncytial Virus (RSV) Replication. Viruses.

[51] Zheng Y., Tang W., Zeng H., Peng Y., Yu X., Yan F., Cao S. (2022). Probenecid-Blocked Pannexin-1 Channel Protects Against Early Brain Injury via Inhibiting Neuronal AIM2 Inflammasome Activation After Subarachnoid Hemorrhage. Front. Neurol..

[52] Box H.J., Sharp J., Pennington S.H., Kijak E., Tatham L., Caygill C.H., Lopeman R.C., Jeffreys L.N., Herriott J., Neary M. (2024). Lack of antiviral activity of probenecid in vitro and in Syrian golden hamsters. J. Antimicrob. Chemother..

[53] Guerrero-Plata A., Casola A., Suarez G., Yu X., Spetch L., Peeples M.E., Garofalo R.P. (2006). Differential response of dendritic cells to human metapneumovirus and respiratory syncytial virus. Am. J. Respir. Cell Mol. Biol..

[54] Hamelin M.E., Yim K., Kuhn K.H., Cragin R.P., Boukhvalova M., Blanco J.C., Prince G.A., Boivin G. (2005). Pathogenesis of human metapneumovirus lung infection in BALB/c mice and cotton rats. J. Virol..

[55] Huck B., Neumann-Haefelin D., Schmitt-Graeff A., Weckmann M., Mattes J., Ehl S., Falcone V. (2007). Human metapneumovirus induces more severe disease and stronger innate immune response in BALB/c mice as compared with respiratory syncytial virus. Respir. Res..

